# Adopting the Concept of ‘Ba' and the ‘SECI' Model in Developing Person-Centered Practices in Child and Adolescent Mental Health Services

**DOI:** 10.3389/fresc.2021.744146

**Published:** 2022-01-05

**Authors:** Christie Attard, Michelle Elliot, Paulann Grech, Brendan McCormack

**Affiliations:** ^1^Division of Nursing, Queen Margaret University, Musselburgh, United Kingdom; ^2^Department of Mental Health, Faculty of Health Sciences, University of Malta, Msida, Malta; ^3^Division of Occupational and Art Therapies, Queen Margaret University, Musselburgh, United Kingdom

**Keywords:** person-centered care, child and adolescent mental health services, rehabilitation, recovery, SECI model, Ba

## Abstract

The concept of knowledge is divided into explicit and tacit knowledge; explicit knowledge refers to the knowledge that can be articulated, written and stored, while tacit knowledge refers to personal experiences, values, beliefs and emotions of an individual. By Nonaka's theory, explicit and tacit knowledge do not lie separately but interact together by interactions and relationships between human beings. Thus, the SECI model is based on the assumption that knowledge is created through the social interaction of tacit and explicit knowledge; known as knowledge conversion. The SECI model is based upon four modes of knowledge conversion; socialization, externalization, combination and internalization. 'Ba' is considered to be a shared platform for knowledge creation. 'Ba' is a shared space, be it physical, mental or a combination of both that serves as a foundation of knowledge creation. Ba involves sharing of tacit knowledge i.e. emotions, feelings, experiences and mental images. It also involves the formation of a collective relationship which is open to the sharing of practices, values, processes and culture. This concept focuses mainly on the individual as a person who holds the knowledge rather than just on the knowledge itself. It aims to create a common space to bring people together where they can dialogue to share and create knowledge. As in the relationships formed in person-centered practices, relationships formed in Ba are based on not just the sharing of objective knowledge but also on sharing values, beliefs, and emotions. It also reflects the formation of a person-centered environment as a basis for person-centered research where healthful relationships with the participants are formed. Furthermore, Ba will aid in creating a sense of connectiveness and dialogue, thus focusing on the idea that the development of new practices is done with others rather than to others. In this article we will discuss how these Eastern concepts can be adapted and used to develop person-centered practices within child and adolescent mental health services, specifically related to rehabilitation and recovery. The concepts of personhood will be discussed, followed by a reflection on current practices adopted when working with children and adolescents.

## Introduction

Health care systems around the world are developing strategies and frameworks to implement person-centered services (1–3). The World Health Organization (2015) describes people-centered health services as services which put people and not diseases at the heart of the health care systems, by encouraging the person to take charge of their care planning rather than being passive recipients of health services. They further describe it as ‘an approach to care that consciously adopts the perspectives of individuals, families and communities, and sees them as participants as well as beneficiaries of trusted health systems that respond to their needs and preferences in humane and holistic ways.' (4).

Education, continuous support, and active participation were highlighted as important factors that lead to the adoption of people-centered health service. The Royal College of Nursing also recognizes the importance of shifting toward person-centered rather than patient-centered care. They simply describe person-centered care (PCC) as focusing the care on the ‘needs of the person rather than the needs of the service' (5). They specify further by saying that PCC involves the active involvement in the care-planning process, and the awareness of the emotional and spiritual well-being of the person in care. By doing this, healthcare professionals ensure that persons receiving care ‘acquire the knowledge skills and confidence' (6) to be able to take charge of their care. This can be achieved by recognizing that everyone has knowledge, values, beliefs, socioeconomic circumstances which makes his/her care unique and different from any other. Santana et al. (7) argues that PCC does not only refer to the care given to the person in care, but also involves the families, caregivers, and the work done in the prevention and promotion areas (7).

The important shift from being a patient to a person, shift the focus from the sickness of a person toward one that looks at the person with a social role away from just the medical diagnosis. Looking at ‘patients' often led to a paternalistic approach to healthcare where ‘healthcare professionals legitimately exercised the governance of people in care through their control of knowledge and the body, while people became subjects of their power-knowledge positions' (8). Focusing solely on the diagnosis, medical assessment, and medical treatments, without taking values and beliefs into consideration may lead to the objectification of people (9). By being person-centered, healthcare professionals are moving away from the ‘objectification' of persons to having a subjective view, the ‘sense of feeling, mood and emotion'(9) is recognized. This is done by developing compassionate care relationships, and focusing on the needs and wants of the person using the services (8). Studies which explored person-centered care explained that PCC resulted in shorter hospital stays, and improvement in health and functional performance (2).

In the area of child and adolescent healthcare, the question is whether services are being person-centered with a focus on the needs, values and beliefs of the child, or family centered with a focus on the needs of the family as a unit with little attention to the young person her/himself. In the development of person-centered therapy Carl Rogers challenged the authoritarian way that parents used with their children, which was also observed during therapy (10). Mostly, practices across the world have shown that parents or main care givers have the active role in the child's care, while the child or adolescent was still given the passive role (11). While the involvement of the parents is essential in the care of children and adolescent, one questions if healthcare practices are acknowledging and giving the children's right to be active participants in their care.

In this paper we will discuss how person-centered practices can be further developed in child and adolescent mental health services (CAMHS), focusing on rehabilitation and recovery of young persons suffering from mental health disorders. We will do this by firstly giving a brief overview about child and adolescent mental health, followed by an exploration of the philosophical underpinnings of personhood as related to children and adolescents. Implications of these underpinnings when working with children will then be discussed. The ‘SECI' model and the concept of ‘Ba' will be introduced. Ultimately these Eastern concepts are further explored in ways to compliment the existing research and person-centered frameworks, specifically related to development of person-centered practices in rehabilitation and recovery services in child and adolescent mental health.

## Child and Adolescent Mental Health

Child and adolescent mental health have been given more importance for the last few years, as most of the time mental health disorders in adults have an antecedent in childhood (12). Unfortunately, there is a trend of increase in mental health disorders or probable mental health disorders in the child and adolescent population. This is highlighted in the NHS (England) findings of a survey carried out in 2020, where it was noted that there was an increase of more than 5% in rates of probable mental health disorders in both boys and girls aged between 5 and 16 years since 2017 (13). Such alarming statistics are also seen on a worldwide level where the World Health Organization (14) reports that 16% of children and adolescents worldwide suffer from a mental health condition. Furthermore, depression is the leading cause of disability worldwide among adolescents, while suicide is the third leading cause of death in adolescents between 15 and 19 years old (14). The global pandemic of COVID-19 for the past year also left a great impact on young people's mental health, with more than half of young people with probable mental health disorders, reporting getting worse during the lockdown periods (13). Anxiety related to the pandemic and sleep problems were also reported by parents and children themselves, furthermore one in ten of 11- to 22-year-olds reported of feeling mostly lonely during the pandemic (13). Children and Adolescent mental health is an important aspect since half of the mental health disorders in adults occur before the age of 14, additionally if mental health conditions are not treated during childhood and adolescence there is a high possibility that they will extend in adulthood causing significant impairment of the social and emotional well-being (13).

Unfortunately, the sector of child and adolescent mental health has not been given the adequate importance when compared to adult mental health (15). It is well-known that child and adolescent mental health is a key area of concern, mainly because it is highly likely that if mental health disorders are left untreated, they can lead to severe difficulties in adulthood. Childhood mental health disorders are often associated with ‘school failure, self-harm, (sexual) risk-taking behavior and serious dysfunction in adult life, thus placing a burden on children, families and communities' (16). Furthermore, it is noted that in the disability adjusted life years (DALYs) calculation the child and adolescent population are severely under-represented (15). This shows a serious neglect on the impact that the mental health disorders can have on the child's overall health and function (16). This often led the services worldwide to focus on the improvement of early intervention rather than focusing on recovery or rehabilitation, leading to services lacking in recovery awareness (17, 18).

Moreover, little attention is given to the expectations or personal experiences of the children and adolescents themselves (19). The views of recovery or rehabilitation are often based on the main care giver and healthcare professionals' perceptions and expectations. Symptom remission in child and adolescent's mental health disorders is most often attributed to time and maturation, rather than personal effort (19). To make the services more focused on effective rehabilitation and ultimately recovery, the focus should be on the child and adolescents' experiences and expectations. Present research still lacks the knowledge of the child and adolescents experiences, and thus it is vital that a better understanding of the child and adolescents experience of the mental health disorder starting from the point of onset, diagnosis, progression and ultimately remission is gained (19).

## Personhood

One of the core philosophical underpinnings of developing person-centered practices, is the meaning of a ‘person'. The different philosophical views offer differing perspectives about the ‘characteristics' that define a ‘person,' which shaped the different theoretical frameworks that practice is based upon, such frameworks will be explored further with a specific relation to children and adolescents.

When we think of a child one would often think of a ‘person who in some fundamental way is not yet developed, but who is in the process of developing' (20). Plastow argued that ‘childhood is, in one sense, a state determined by the societal, political, ideological, and even clinical discourse that prevail' (21).

Schapiro points out that when a person is called a child, an assertion and an assumption is made that s/he lacks certain biological features which are attributed to adults (20). Childhood can be described as a ‘condition which prevents the person from achieving autonomy' (20), yet not even adults are able to develop full autonomy. These thoughts often lead adults to show paternalistic behaviors, but our obligation as adults is to treat children as ‘practical agents' with a potential of finding reasons into actions (20). Thus, rather than imposing decisions (as adults), the best decisions should be discussed in view of the child's opinion and wishes.

In his philosophy Immanuel Kant and his followers distinguishes between ‘persons' and ‘things,' he describes that the main difference between the two is that persons are ‘rational beings' (22). Christine Korsgaard further defines this, as the ability to base decisions upon reason rather than on what is desirable and what contributes to ‘survival and instinctual satisfaction' (23). Joshi argues that if we attribute such definition to newborn babies, then they can't be considered as ‘persons' since they act by instinct rather than being rational (22). He further argues that such attributes start to develop when the child is between the ages of three and four, but there is no clear indication when the child will reach this milestone and therefore meet Korsgaard's definition of being a person (22). Engelhardt argues that persons are the beings that possess ‘moral rationality in the sense that of being able to appreciate that actions can be tied to state of blameworthiness or praiseworthiness' (24). Engelhardt states that since infants and persons with intellectual disabilities lack such moral rationality, they cannot be called persons, but are referred to as ‘human nonpersons' (24). He further discussed that persons have a moral obligation toward infants since they can be considered as the ‘future person' (24).

As there is a clear distinction between infants and children becoming a person, developmental psychology investigated the transition of how infants develop morality. This process is described as ‘multifaceted and gradual' (22). Having morality is closely associated to developing moral obligation, as being aware of what is morally wrong will result in having a moral obligation of not to do it (25). To explore the process of the development and origins of moral obligation, Piaget focuses on the child's ability to respect the rules, initially done through play. To initiate this, Piaget describes morality ‘as a system of rules, and the essence of all morality is to be sought for in the respect which the individual acquires for these rules' (26). He further described this process as being ‘a continuum which cannot be cut into sections' (26). Thus, Piaget (26) developed the ‘stage theory' approach which later was further developed by Kohlberg to describe the attainment of morality in childhood and therefore the development of moral judgement (27). By this theory it is assumed that by ages 6 and 10, outcome-alone based concepts of morality are fully replaced by intent-based concepts of morality (28). Cushman et al., also agreed that the process of developing moral judgment is gradual and usually happens between the ages of 4 to 6 years old (28). They describe the process of attaining moral judgement as being a two-process system; outcome-based process and intent-based process. Younger children perceive actions to be more punishable depending on the degree of damage (outcome-based), but as they get older the intention behind the damage is taken in consideration (intent-based) (28). Cushman et al., concluded that around the age of six, the child generally acquires an intent-based process of moral judgment (28). By following this line of thought it can be concluded that the process by which children become ‘persons' in the Kantian sense is a gradual process that differs from one child to another.

Conversely, Hamlin et al., argue that 6 and 10-month old infants were able to judge and show preference to individuals based on their actions, i.e. prefer individuals who helped another and neutral individuals i.e. ones who did not cause harm (29). Furthermore, Smetana concluded that at the age of 2 ½ years children were able to distinguish their judgements based upon moral events or social conventional events (30). In their study Nobes, et al., challenged Piaget's findings that children's moral judgements are based on outcomes rather than on intentions (31). They determined that children as young as 3 years old were able to base their judgements taking outcome, negligence, and intention into consideration (31). By using a narrative approach, Komolova et al., concluded that the ‘emergence of psychological and relational conceptions of persons' start at an early age, also showing that young children had an ability ‘to integrate relational and psychological understandings of persons' (32). Conversely, taking in consideration brain plasticity which is severely dependent on various factors including genetics, pre-natal stress, and the environment the child is brought up in, the stages of brain development are most likely to vary between all children (33). Thus, the attainment of moral judgment can take place at different ages depending on various genetic, and pre and post-natal factors.

Following Korsgaards' definition of being a person and Piagets' and Kohlberg theories of attaining morality, one would question the personhood of children and adolescents who live with conditions which may affect their development. When considering persons with autism spectrum disorder (ASD), it is noted that while they are able to develop a basic form of moral judgement by relying on the emotional and external aspects of the moral case, they are unable to develop intent-based moral judgment due to the inability to understand cognitive aspects. Although children with ASD were shown to be able to interpret actions during pre-school years (34) (following the same developmental pattern as children who do not experience ASD), evidence still shows that this understanding is not developed into intent-based moral judgment (35). Thus, following the process of developing moral judgement as discussed by Cushman et al., children and adolescents with ASD lack the ability to fully develop moral judgment and therefore not be considered as persons in the Kantian sense.

The development of morality is determined by the emotional and social development, which is known to be affected in children with attention deficit hyperactivity disorder (ADHD). The development of empathy and guilt seem to be the most particularly affected, thus risking ‘damaging the moral reasoning capabilities of an individual' (36). Furthermore, the difficulty faced by children with ADHD in developing socialization skills may also pose a risk on the development of morality (37). Thus, children experiencing ADHD who lack this development are not considered as persons in the Kantian sense. One may question that given that ‘reparative measures are possible, especially through therapeutic interventions found in cognitive-behavioral therapy (CBT)' (36), when can a child with ADHD be considered as a ‘person', and who is to decide.

McCormack and McCance argue that we should not attribute personhood solely to the cognitive and rational abilities (38). They described that ‘Connecting with an innate sense of ourselves as human beings with feelings, emotions, thoughts and desires is an essential component of being a person and de facto having personhood' (38). Young describes personhood from a psychological point of view and claims that personhood cannot be explained without ‘considering the individualized developmental trajectory each person traverses in creating her/his sense of self, identity, self-esteem, values, and abstract logical structures applied to contextual matters and planned for ensuing embeddings' (39). This was shown in the study by Komolova et al., where they attributed both rational and psychological themes to the narratives of children and adolescents (32). Mascolo also argued that to be able to describe and understand persons we must move beyond distinction between objectivity and subjectivity toward the idea of intersubjectivity (40). Mascolo describes persons as ‘self-conscious, agentive, relational animals who, by virtue of their capacity for symbolism and intersubjective engagement, act on the basis of their identification with social systems of meaning and value' (40), thus by this definition the mind and body, and the ‘mental' and the ‘behavioral', do not need to be distinguished.

These different perspectives of the child as a ‘person' inform the practice of mental health professionals and other professionals who work with children and young people. For example, the view that young people lack moral judgment can lead to decision making done solely by adults. Whereas focusing on attributes other than cognitive and rational abilities, professionals would try to consider more the emotions, thoughts, and desires of young people, and therefore they will be active players in their care.

## Working With Children

The UNICEF conventions (1989) described the child as being any person under the age of 18 years old. One of the basic children's rights as agreed by UNICEF is that ‘children have the right to give their opinions freely on issues that affect them. Adults should listen and take children seriously' (41). One questions if professionals working with children and adolescents are truly ensuring that this right is met. Professionals working with children mainly follow two discourses: that the young person needs protection and support from an adult and secondly and closely related is that the child needs adult instruction, control and even punishment (42). Professionals tend to seek the advice of the main caregivers to develop a way forward in the care of the young persons, when this is lacking the role is taken upon the healthcare professionals involved. This puts young people in a vulnerable position in fact they are at a risk of abuses of power, similar to any other marginalized group (42). Matthews goes further by arguing that ‘Unlike other marginalized groups children are often not in a position to enter into dialogue with adults' (43), and their representation is still lacking in decision-making processes. This is even more evident in the healthcare system where their involvement in decision making is ‘almost none' (44). Children are taken to the physician usually upon the decision of the adult, and when at the physician the dialogue is mostly made with the adult rather than the child, ‘viewing the child primarily as the bearer of pathology' (44). This is common practice, mainly because legally, children younger than 16 years old should not be given treatment without parental consent (45, 46). Such a decision is based upon the argument that minors are ‘dependent beings who lack capacity' to make mature independent choices (47). Redding argued that parents and healthcare professionals do not always act in the best interest of the child especially in the mental health context, due to many reasons like scapegoating, burn out of parents, and stigma (48). Thus, such decisions should be made in collaboration with the child, rather than given to the child. That is, it should be ensured that the child's need and desires are heard, and final decisions are communicated and discussed effectively with the child.

## The Seci Model

The SECI model was first introduced by Ikujiro Nonaka, and later expanded by Nonaka and Hirotaka Takeuchi. The initial aim was for the model to be used in the context of business and organization expansion but since then it has been adapted to other areas including education and healthcare (49, 50). This model of knowledge creation is based upon Japanese epistemological perspectives, which differ from Western views as they place more value on ‘the embodiment of direct, personal experience' (50). Nonaka explained that knowledge is a continuous interaction between tacit and explicit knowledge that results in the generation of new concepts and ideas (51). In healthcare systems, while knowledge sharing is essential to ensure patient safety, it is vital that knowledge creation is fostered through daily activities and external acquired knowledge (52).

The concept of knowledge is divided into explicit knowledge and tacit knowledge; explicit knowledge refers to the knowledge that can be articulated, written and stored, while tacit knowledge refers to personal experience, values, beliefs and emotions of an individual (53). The interactions between the two types of knowledge at every organizational level and by every member of the organization can lead to knowledge creation (54). This process lies on a continuum; ‘When an individual's tacit knowledge is shared with another person it becomes explicit knowledge, and when this is merged with other explicit knowledge it becomes new explicit knowledge, which in turn can then be converted into the tacit knowledge of another (or the same) individual and thus link with the subsequent conversion process' (55). By this theory, explicit and tacit knowledge does not lie separately but interact together in the ‘creative activities of human beings' (56). Thus, the SECI model is based on the assumption that knowledge is created through social interaction of tacit and explicit knowledge; known as knowledge conversion. Nonaka and Tekeuchi explained four modes of knowledge conversion:

1. Socialization: Tacit knowledge 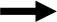 Tacit knowledge: Process of sharing experiences, thus creating new tacit knowledge. This can be done using language, observation, imitation and practice. Leads to the formation of sympathized knowledge: shared mental models and technical skills.
2. Externalization: Tacit knowledge 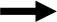 Explicit knowledge: Articulating tacit knowledge into context, thus forming hypothesis, concepts, or models. Leads to conceptual knowledge.
3. Combination: Explicit knowledge 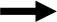 Explicit knowledge: Exchange and combination of knowledge using documents, meetings, virtual meetings, and phone conversations. Outputs Systemic knowledge: prototype.
4. Internalization: Explicit knowledge 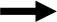 Tacit knowledge Shared mental models or technical know-how, formed after the experiences of the above three steps. Gives rise to operational knowledge: policy implementation (56).

This process of knowledge creation (as represented by the SECI model) takes place within the concepts of ‘Ba', which is a shared platform of knowledge creation. Knowledge creation is represented as a spiral as ‘innovation emerges from the spiraling continuity of this conversion process' (54). Following this model can be an essential process for knowledge creation and innovation, Easterby-Smith et al., states that if interorganizational transfer of knowledge is encouraged, it often results in strengthened organizations knowledge and innovative capabilities (57).

## The Concept of Ba

‘Ba' describes a field or space where human interactions take place and relationships are formed (50). It is a shared space, be it physical (office), mental (shared ideas or experiences) or a combination that serves as a foundation of knowledge creation. Ba involves sharing of tacit knowledge i.e. emotions, feelings, experiences and mental images. It also involves the formation of a collective relationship which is open to the sharing of practices, values, processes, and culture. Nonaka emphasizes that ‘care, love, confidence and responsibility are required.' (58). Ba provides a context for internalization of knowledge and catalyses reflection which is then transformed into action. This concept mainly focuses on the externalization of tacit knowledge, this type of knowledge is ‘intangible, unbounded, and dynamic' (59). The main focus is on the individual as a person who holds the knowledge rather than just on the knowledge itself. It aims to create a common space be it physical or virtual to bring people of the organization together where they can dialogue to share and create knowledge and thus ultimately leading to innovation.

A ba can be formed both formally and informally. Nonaka and Takeuchi argue that interactions that takes place in an informal environment (e.g., a café) can be considered as informal ba, as during this time people may be sharing opinions and concerns that may lead to new insights (50). In formal ba (e.g., a formal workshop), persons with a shared goal will closely interact with one another, thus forming relationships that will in turn lead to the understanding of each other's views and values. Furthermore, a ba can also be a place with ‘embedded' (50) knowledge, for example tacit knowledge about person-centered practices in a CAMHS unit can be gained by spending time in the unit and interacting (forming relationships) with staff and young persons using services. Thus, in this example the CAMHS unit is the Ba as it is the place where personal interactions, sharing of experiences, and values, and therefore sharing of knowledge is being fostered. In this sense, ‘a ba is both an incubator of knowledge as well as a container of knowledge' (50).

As in the relationships formed in person-centered practices, relationships formed in Ba are based on not just the sharing of objective knowledge but also on sharing values, beliefs, and emotions. It also reflects the formation of a person-centered environment as a basis for person-centered research where healthful relationship with participants are formed (60). Furthermore, Ba will aid in creating a sense of connectiveness and dialogue between all participants, thus focusing on the idea that the research and innovation is done with others rather than to others (61).

## The Seci Model, Ba and Person-Centeredness

When the SECI model was adopted in Western health research it provided the opportunity to patients to externalize tacit knowledge, after going through all stages of the SECI models the doctors ended up with more knowledge which ultimately resulted in care which was more sensitive toward the person's needs (58). The first stage of the ‘SECI' Model, emphasizes the importance of gathering tacit knowledge from everyone involved in the organizational system. Persons are encouraged through Ba to share their personal knowledge, be it being children and adolescents, their main care givers or the healthcare professionals providing the service. Although gathering tacit knowledge is not as straight forward as it is for explicit knowledge it offers ‘highly subjective insights, intuitions, and hunches' (56), and thus it is very personal. This makes the process of knowledge creation a very individual process for all involved which ultimately leads to personal and organizational (service) self-renewal. As it is shared, the knowledge (personal experiences, mental models, beliefs) then becomes explicit knowledge for all the group members, who can then draw on that explicit knowledge to develop new knowledge. Sharing this explicit knowledge will form new tacit knowledge for each person involved. Although the process considers each individual involved, a very important factor about it is that it continuously involves the sharing of group knowledge, which ‘results in a transcendence or connection between individuals' (62). The socialization step of the model in particular, require the mutual participation of all persons involved (63), therefore fostering co-operation and relationships with others. By creating Ba within a system, ‘environments that are able to foster rich relationships between individuals and encourage innovation' would be created (62). This is essential to be able to form a care environment that is conducive in developing person-centered practices.

This theory of knowledge creation is based upon the Japanese intellectual tradition. One of the key distinctions in this tradition is the ‘oneness of body and mind' (56). It values the importance of acquiring knowledge from the entire personality (i.e. body and mind), and values the embodiment of direct, personal experiences. This is congruent with the idea of personhood as presented by McCormack and McCance where it was argued that personhood should not only be attributed to the cognitive and rational abilities (38). Another distinction in the Japanese tradition is the ‘oneness of self and other' (56), which refers to the value given to the ‘interaction between self and others' (56). Just like in the formation of person-centered practices, an emphasis on having healthful relationships with others is made, where things are conceptualized by relating to others.

## Adaptation in Child and Adolescent Mental Health Recovery

Child and adolescent mental health recovery mostly follow prominent recovery models, but such models are formulated for the adult's population. Thus, important factors like developmental factors related to child and adolescent mental health were not taken into consideration in such models (64). Recovery is considered to be ‘a unique, non-linear, personally driven entity' (64), but when considering young persons recovery, research shows the importance of involving the main care givers and professionals involved in the care of the young person (65). The important role of parental involvement is continuously highlighted, this is mainly due to the need of the parents who will generally enable engagement to the services (64). Furthermore, it is vital that everyone involved understands the personal experience of the whole process of the young person involved. The theory of youth mental health recovery by Kelly and Coughlan ‘suggests that due to developmental factors, youth mental health recovery occurs within the ecological context of complex hierarchal interconnected social relationships' (66). These relationships include all persons that in some way or another have a relationship with the young person involved i.e. parents, healthcare professionals, educators, friends and the society as a whole. The young person is considered to be at the top of the hierarchy as they are personally experiencing the mental health disorder and the process of recovery. Due to the importance of such relationships, it is vital that throughout the recovery process connections are developed and fostered. These connections will enable the young person to experience ‘hope, acceptance, positivity and normality' (66), and thus promote resilience and a sense of control over one's mental health.

These important factors that make up the recovery model, are congruent with the ones present in the SECI model and the concept of Ba. By following the socialization stage of the SECI model, the young persons tacit knowledge would be considered, thus giving the opportunity to the young person to fully share his/her personal experience. But it is also essential that tacit knowledge is gained from all the persons involved, thus involving the persons involved in the hierarchy of social relationships as explained by Kelly and Coughlan (66). Therefore, as with the socialization stage of the SECI model, the aim of this process is ‘the sharing of the tacit knowledge (56). During the externalization and combination stages of the SECI model, recovery-oriented interventions will be developed. These will be based solely on the tacit knowledge gained in the socialization stage, thus being highly personal and specific to the young person's needs, whilst also taking into account the wishes and values of main givers and young person. Such consideration is essential to empower the young person to be active participants in their recovery process, whilst also garnering hope to the parents (18). During the internalization stage the developed interventions will be implemented by the multi-discplinary team, this is done while still fostering supportive relationships through Ba. These factors are all associated with significant improvement in rehabilitation programs and therefore more improvement toward recovery (67).

By adopting Ba throughout the process, an opportunity of connection between every person involved will be created, thus creating a sense of safety, and understanding which is essential for the young person involved. This sense of connection can be crucial in the recovery process as the young person will ‘experience hope, acceptance, positivity and normality' (59), thus enhancing resilience and a sense of control over one's mental health. These factors are all essential in promoting child and adolescent mental health recovery. By encouraging communication between all professionals, parents, and young person throughout the SECI model positive connections will be promoted throughout, which will in turn improve communication. understanding and support and ultimately promote recovery (64).

By following the SECI model and creating Ba within healthcare services, healthcare professionals will be personally involved in the whole process and will be able to offer individualized support to all persons involved and form positive connections. These connections are not only formed with the young person but also with the main care givers, school, and other professionals involved, thus ensuring a holistic person-centered approach toward care. Such involvement is vital as mental health recovery in children and adolescent mental health may be dependent not only on the young person him/herself but also on all the persons directly involved in the care (18). This model can be adapted either on an individualized level, thus following it to promote individualized interventions that promote recovery, or at a generalized level to develop mental health recovery models which are person-centered and specific to the needs of the young persons.

**Figure 1:**
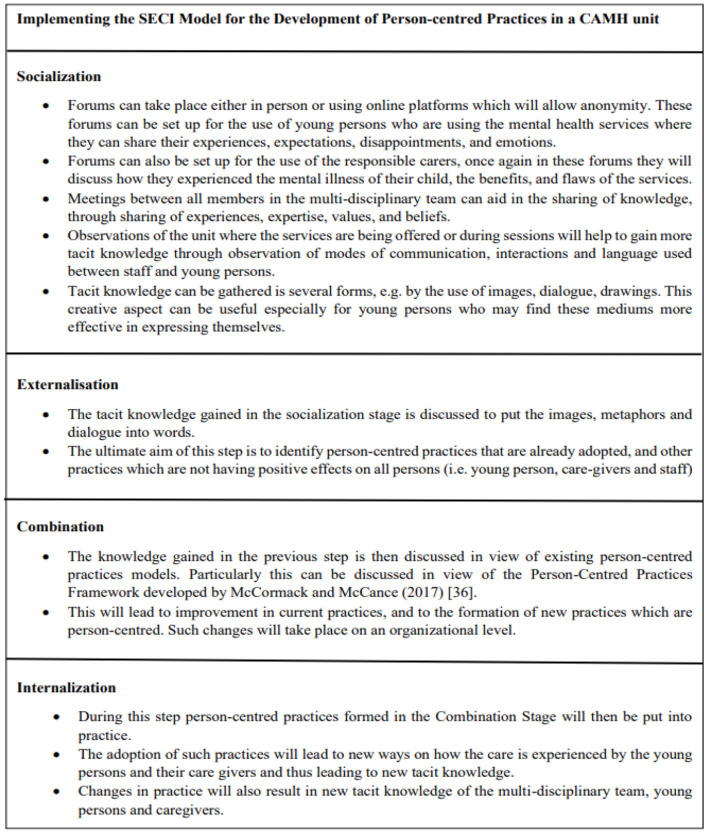
Application of the SECI model in the development of person-centered practices in a CAMH unit.

**Figure 2:**
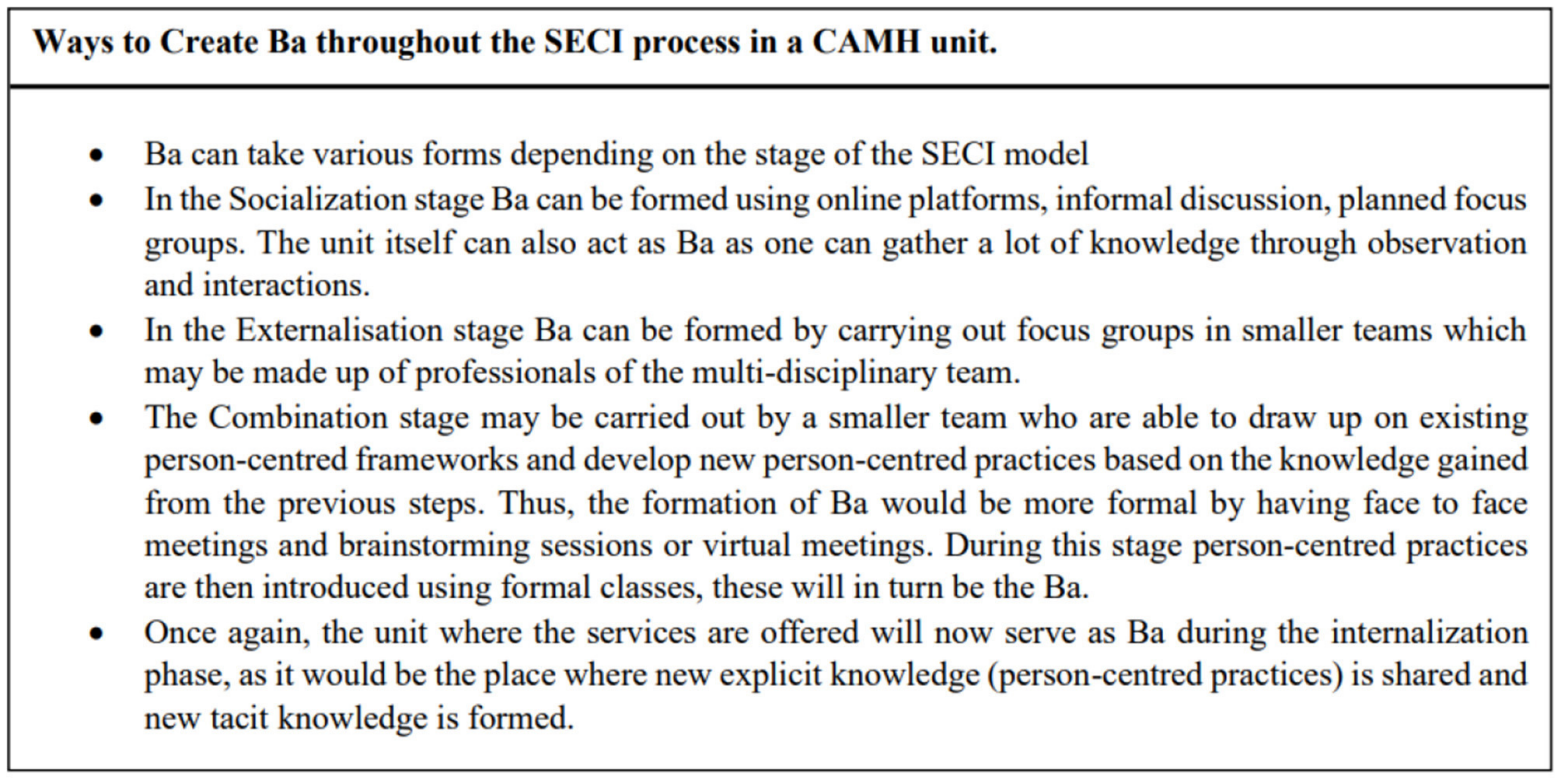
Approaches to creating Ba in a CAMH unit.

Figure 1 (above) presents a case example of how the SECI model can be adapted on an organizational level in a CAMHS unit. In the socialization stage opportunities are created for gathering tacit knowledge, this will include the experiences of the young persons and how the mental illness and the road to recovery have affected them. During this stage the main care givers are also given importance since they play a significant role in the care of children and adolescents. Tacit knowledge is also gathered from the professionals who work in the unit, through a variety of approaches to conversation and dialogue, and through observation. This gathered tacit knowledge can be insightful in ways that show how person-centeredness is experienced (or not) by all persons. Ba can be created in several forms, as explained in Figure 2 (above), it can be both formal and informal and will involve the young persons, caregivers, and professionals. Ba can also be created virtually, for example by creating online forums. This can be particularly effective when working with young people, as such platforms are easier to access, and the young person can remain anonymous when sharing his/her experience. Different mediums of expression can also be used, for example the use of metaphors, drawings, and images, to facilitate the sharing of tacit knowledge. In the externalization stage, these are then turned into words. This stage can be carried out by smaller groups who will identify the person-centered practices that are adopted and point out specific needs for improvement. During this stage a formal Ba can be created by forming team meetings or focus groups. More than one team can be created, and Ba can be both virtual or in-person. The explicit knowledge gathered would then be discussed in the context of a formal framework for developing person-centered practices, such as that by McCormack and McCance (38). In this combination stage, the improvement and new person-centered practices can be developed by drawing upon the explicit knowledge gained throughout the model and the explicit knowledge of the Person-Centered Practice Framework. This stage is carried out by forming Ba between smaller working groups through face-to-face or virtual meetings. These will then be shared with the other professionals through formal meetings, seminars, and workshops. The final stage of the SECI model is the implementation of person-centered practices. This in turn create new tacit knowledge for the professionals, young persons using the services and main care givers. This process of knowledge creation can be possible when all different perspectives and views are given equal importance, which will in turn lead to the formation of relationships with shared values, beliefs and emotions (50).

## Conclusion

Healthcare systems around the world are striving to develop and implement person-centered practices. The development of such practices can be challenging especially when working with children and adolescents. In this area one may still question if care is focused on the child/adolescents wishes and needs or the decisions made by the adults responsible for their care, and research related to rehabilitation and recovery is particularly lacking (66). By following the SECI model and introducing the concept of Ba we may develop such person-centered practices which are recovery oriented while ensuring that each person's experiences, values, and beliefs are given value throughout the process. It is important to note that further research on specific methods of implementation of each stage of the SECI model, and the incorporation of person-centered frameworks is essential.

## Data Availability Statement

The original contributions presented in the study are included in the article/supplementary material, further inquiries can be directed to the corresponding author/s.

## Author Contributions

CA designed the original study idea, and led the theoretical development with contributions from BM, PG, and ME. All authors designed the methodology. CA wrote the first draft of the manuscript while BM, PG, and ME contributed to redrafting and approved the final version.

## Conflict of Interest

The authors declare that the research was conducted in the absence of any commercial or financial relationships that could be construed as a potential conflict of interest.

## Publisher's Note

All claims expressed in this article are solely those of the authors and do not necessarily represent those of their affiliated organizations, or those of the publisher, the editors and the reviewers. Any product that may be evaluated in this article, or claim that may be made by its manufacturer, is not guaranteed or endorsed by the publisher.
